# Comprehensive Integration of Genome-Wide Association and Gene Expression Studies Reveals Novel Gene Signatures and Potential Therapeutic Targets for *Helicobacter pylori*-Induced Gastric Disease

**DOI:** 10.3389/fimmu.2021.624117

**Published:** 2021-02-24

**Authors:** Mohamed Tarek Badr, Mohamed Omar, Georg Häcker

**Affiliations:** ^1^ Faculty of Medicine, Institute of Medical Microbiology and Hygiene, Medical Center—University of Freiburg, Freiburg, Germany; ^2^ IMM-PACT-Program, Faculty of Medicine, University of Freiburg, Freiburg, Germany; ^3^ Department of Pathology and Laboratory Medicine, Weill Cornell Medicine, New York, NY, United States; ^4^ BIOSS Centre for Biological Signalling Studies, University of Freiburg, Freiburg, Germany

**Keywords:** *Helicobacter pylori*, gastritis, genome-wide association study, transcriptomics, gene-signature, immune response, multi-cohort analysis, gastric cancer

## Abstract

*Helicobacter pylori* is a gram-negative bacterium that colonizes the human gastric mucosa and can lead to gastric inflammation, ulcers, and stomach cancer. Due to the increase in *H. pylori* antimicrobial resistance new methods to identify the molecular mechanisms of *H. pylori-*induced pathology are urgently needed. Here we utilized a computational biology approach, harnessing genome-wide association and gene expression studies to identify genes and pathways determining disease development. We mined gene expression data related to *H. pylori-*infection and its complications from publicly available databases to identify four human datasets as discovery datasets and used two different multi-cohort analysis pipelines to define a *H. pylori-*induced gene signature. An initial *Helicobacter*-signature was curated using the MetaIntegrator pipeline and validated in cell line model datasets. With this approach we identified cell line models that best match gene regulation in human pathology. A second analysis pipeline through NetworkAnalyst was used to refine our initial signature. This approach defined a 55-gene signature that is stably deregulated in disease conditions. The 55-gene signature was validated in datasets from human gastric adenocarcinomas and could separate tumor from normal tissue. As only a small number of *H. pylori* patients develop cancer, this gene-signature must interact with other host and environmental factors to initiate tumorigenesis. We tested for possible interactions between our curated gene signature and host genomic background mutations and polymorphisms by integrating genome-wide association studies (GWAS) and known oncogenes. We analyzed public databases to identify genes harboring single nucleotide polymorphisms (SNPs) associated with gastric pathologies and driver genes in gastric cancers. Using this approach, we identified 37 genes from GWA studies and 61 oncogenes, which were used with our 55-gene signature to map gene-gene interaction networks. In conclusion, our analysis defines a unique gene signature driven by *H. pylori-*infection at early phases and that remains relevant through different stages of pathology up to gastric cancer, a stage where *H. pylori* itself is rarely detectable. Furthermore, this signature elucidates many factors of host gene and pathway regulation in infection and can be used as a target for drug repurposing and testing of infection models suitability to investigate human infection.

## Introduction

*Helicobacter pylori* colonizes the stomach of approximately half of the world’s human population. This colonization is mostly asymptomatic, but in some cases an immune response is initiated that may cause chronic inflammation of the gastric mucosa and can lead to various severe conditions such as peptic ulcer disease and gastric cancer (1, 2). *H. pylori* antibiotic resistance, a major cause of failure of eradication therapy, is increasing, and the World Health Organization (WHO) has listed *H. pylori* accordingly among bacteria that urgently need new therapies. This highlights the need for new efforts to understand the mechanisms underlying *H. pylori* transmission, colonization, pathogenesis, and treatment failure. The development of gastritis and subsequently gastroduodenal ulcer diseases and cancer is a multifactorial process, and both the environmental and genetic background of the patient contribute (3). Previous Genome-wide association studies (GWAS) have been able to identify genetic polymorphisms in the toll-like receptor (TLR) locus that correlate to patients’ *H. pylori* seropositivity. Furthermore, these patients showed high expression levels of TLR1 (4). Other studies have identified an association of polymorphisms in the TLR5 gene with atrophic gastritis (5) as well as other autoimmune reactions (6). These studies hint at a complex regulatory network for disease progression during *H. pylori-*infection. This network may be mostly controlled through environmental factors, bacterial pathogenic antigens such as CagA or VacA, and also the patients’ genetic background and immune response to the infection. Further gene expression studies from human patients and experimental models have elucidated many of the molecular mechanisms relevant to *H. pylori* pathogenicity and the pathways related to the various disease stages. However, their results remain indecisive as they show a variable picture, most likely due to low sample numbers in individual studies or variations in disease stage and severity in analyzed samples (7, 8). A powerful model to study a tissue and cell specific reaction to *H. pylori* especially at the different stages of the pathology is the use of cell lines or animal models. Such studies have elucidated many factors that contribute to disease pathogenesis. The problem of limited reproducibility for some of the identified gene signatures in human patients however remains (9), and contradictory results depending on the cell line and infection conditions have been reported (10). The use of adenocarcinoma cell lines is also limiting because many of the primary cell transitions would be hard to detect, and the suitability of cellular systems to imitate the host’s reaction to the infection is difficult to predict purely on the basis of such biological studies.

Recently developed tools and databases of GWA studies have enabled the collective analysis of diseases’ genetic variants across many samples, which facilitates the discovery of the molecular bases of this association between various diseases and genetic polymorphisms (11–13). This is particularly relevant for reconstructing upstream signals that lead to disease specific gene signatures. A practical approach towards heterogenic disease gene signatures that may develop due to technological bias or experimental factors is to reanalyze these studies with different computational and statistical methods that compensate for these differences. This approach has been very successful in harmonizing the analysis of different studies, by allowing the use of large sample numbers and thereby permitting the identification of novel markers for various diseases (14–16). New associations between different pathologies such as infection and autoimmunity have also been found through such approaches (17–19). Combining genomic and transcriptomic analyses can help better understand the molecular pathways and processes associated with *H. pylori* infection and define disease signatures associated with different stages of disease development. Such a tool could improve patient diagnosis and treatment efforts. Furthermore, comparing gene signatures of human patients with signatures obtained from different cell lines might close the gaps between both signatures and permit assessment of the suitability of cell lines for investigating phases and pathways in infection and disease.

## Methods

### Collection of Gene Expression Data

Collection of the meta-analysis data was carried out by searching public expression databases (NCBI GEO and Array Express) (accessed August 2020). For the GEO query we used the following search terms: “Helicobacter pylori” and the filters [organism (Homo sapiens)], study type (expression profiling by array), entry type (Dataset/Series). The Array Express query was executed using the following search terms: “Helicobacter pylori” and the filters [organism (Homo sapiens)], experiment type (array assay). Initially 55 entries from GEO and 34 entries from Array Express were retrieved. Duplicates and irrelevant studies were excluded, and 32 studies remained. These studies were further refined using the following inclusion criteria to arrive at the four final studies as discovery and six other studies that were assigned for validation. We included only studies that had analyzed gene expression in gastric biopsies or relevant human cell lines. Only datasets with available raw data were included. For the human samples, uninfected healthy controls had to be available in each dataset. The patients’ *H. pylori* infection status had to be known, and we accepted studies where at least one of the following diagnostic tests for *H. pylori* had been performed: rapid urease test, culture, serologic analysis, histopathological analysis. For cell line studies, we only included experiments from wildtype cell lines infected with wildtype *H. pylori* (host cell or bacterial mutants and other *Helicobacter* species were excluded). Studies including gastric organoids were not included due to different culture conditions in comparison to standard cell lines. Experiments using infection times between 16 and 24 h were included; studies using longer or shorter infection periods were excluded to ensure reasonable comparison. For adenocarcinoma, normal and tumor tissue had to be available in the same study to be considered. The database-search followed the Preferred Reporting Items of Systematic reviews and Meta-Analyses (PRISMA) statement and is documented in the PRISMA Flow Diagram (20) (**Supplementary File 1**). Only datasets with available raw data were included. After a thorough search and excluding datasets as specified above, four datasets for Human samples (GSE27411, GSE60427, GSE60662, and GSE5081) and four cell line infection datasets (GSE60661, GSE70394, GSE74577, and GSE74492) (7, 21–27) were selected for further analysis. A total of 98 human samples were considered for downstream analysis of the discovery steps, containing data from 72 helicobacter-infected/gastritis/atrophy/metaplasia patients, and 26 healthy controls. Two datasets detected through the search process with gastric adenocarcinoma (E-MTAB-1440 and GSE65801) were collected for the validation process (28, 29). A second search was performed to detect further gastric cancer datasets for the validation process. GEO was mined using the following search terms: “gastric cancer” and the filters [organism (Homo sapiens)], study type (expression profiling by array). This search yielded 280 entries, which were vetted to detect datasets having cancer and normal tissue, a sufficient number of samples, and raw expression data. Ten datasets matching our criteria were chosen to further validate our gene-signature (30–37).

### Determination of *Helicobacter pylori*-Induced Pathologies Score and Validation in Cell Line Model

We used R programming language (version 4.0.2) (38) and the “MetaIntegrator” package (39), which utilizes a gene expression meta-analysis workflow described by Haynes et al. (40). In summary, the MetaIntegrator approach computes a Hedges effect size for each gene in each dataset. These effect sizes are then pooled across all datasets using a random-effect model by assuming that results from each study are drawn from a single distribution and that each inter-study difference is a random effect. The approach computes the log sum of p-values that each gene is up/down-regulated, then combines the p-values using Fisher’s method and finally performs Benjamini-Hochberg false discovery rate (FDR) correction across all genes (41). In our analysis, a gene is considered to be differentially expressed if the absolute value of its effect size is greater than zero, the FDR is less than 5% across all training datasets and it is significantly up/down-regulated in all of the four training datasets with a heterogeneity P-value cutoff of 0.05 (42). To optimize the initial gene signature, we performed a Forward Search process by taking the initial gene set, adding one gene at a time and calculating the weighted Area Under the ROC curve (AUC) resulting from the addition of this gene. Weighted AUC is calculated as: W.auc=S.auc×n, where W.auc is the weighted AUC, S.auc is the sum of AUC of each dataset and n is the number of samples in this dataset. This process is repeated for each gene until the stopping threshold (0 in our case) is reached and the final set of genes will be those that contributed the most to the weighted AUC. We tested performance and consistency of the original gene signature in four independent cell line datasets (GSE39919, GSE70394, GSE74577, and GSE74492). All discovery steps were conducted on the training datasets only.

### Integrative Pathway Analysis

Functional Enrichment analysis for the original MetaIntegrator signature was performed using the Enrich R package against the following databases: GO Biological Processes (GO BPs), GO Molecular Functions (GO MFs), GO Cellular Components (GO CCs), and KEGG. Upstream signaling pathways were extracted using the Signaling Pathway Enrichment using Experimental Datasets (SPEED) web-tool (43). Enrichment for upstream pathways using a list of either upregulated or downregulated genes was tested using the Bates distribution test. In comparison with pathway membership based methods such as Reactome (44) and gene ontology, SPEED offers some advantage due to its ability to infer causative upstream signals. Its overall performance is compatible with GSEA when using the Bates test (43).

### Identification of Differentially Expressed Genes in Individual and Collective Datasets Using Limma and NetworkAnalyst

The datasets were retrieved from the NCBI GEO database using the GEOquery R package (45). Processing of individual datasets was carried out using the limma R package (46). Human gastric biopsies datasets were normalized using log2 transformation and genes with an adjusted p-value of < 0.05 using the Benjamini–Hochberg method were considered for downstream analysis. Different gene IDs were converted to the official gene symbols or Entrez IDs either through the AnnotationDbi R package (47) or DAVID (Database for Annotation, Visualization, and Integrated Discovery) (48). Meta-analysis of human samples was conducted with the web-based tool NetworkAnalyst (49, 50). For each individual dataset we carried out Log2 transformation with autoscaling and inspected possible outliers using principle component analysis (PCA). The individual analysis of each dataset was carried out using the Benjamini–Hochberg’s False Discovery Rate (FDR) with cut-off p-values of <0.05. The microarray chip identifiers were annotated to other suitable Gene IDs including Entrez Gene identifiers, and datasets were merged after annotation. A suitable identification condition for each sample was assigned where only healthy uninfected samples were assigned to the control group and all other conditions (*H. pylori*-infected, gastritis, erosions, atrophy, metaplasia) were assigned to the case group. To ensure unbiased comparative analysis of the different datasets, the batch effect was adjusted through the ComBat batch effect method integrated in NetworkAnalyst and was investigated before and after adjustment through principle component analysis. The size effect method was used to identify DEGs between the cases and controls. Cochran’s Q test was used to estimate the statistical heterogeneity to identify the most suitable effect size model between the fixed and random effects models (FEM and REM). Depending on the Cochran’s Q test results REM was used to identify DEGs, which usually gives more conservative but reliable results. A discovery significant value of <0.05 was used to identify DEGs. NetworkAnalyst integrated visualization tools were used to create heatmap of DEGs. Genes were clustered using single linkage method.

### Identification and Validation of the Refined Gene Signature

Intersection between the MetaIntegrator and NetworkAnalyst gene signatures was carried out through InteractiVenn (51). The refined 55-gene signature was tested in 12 independent gastric cancer datasets. We used both the Area Under the Receiver Operating Characteristic Curve (AUC), and the Area Under the Precision-Recall Curve (AUPRC) as evaluation metrics.

### Genome-Wide Association Studies Data Collection and Analysis

Collection of GWAS data was carried out by searching the NHGRI GWAS catalog database using the keyword “Helicobacter pylori”, “Gastritis”, or “Gastric cancer” (52). In total 64 SNPs were considered for further analysis. The corresponding genes were identified, and the nearest upstream and downstream genes were selected for intergenic variants. Genetic variants were annotated using the HaploReg v4.1 tool (53). Further gastric cancer driver genes were gathered from the IntOGen database (54). In total 61 genes were discovered through the database research.

### Hub Genes Network Analysis

Protein-protein interaction (PPI) networks were generated using the IMEx Interactome innateDB database (55). A gene list with the 55-gene signature, the 37 GWAS genes, and the 61 gastric cancer driver genes was used for the analysis. A first-order PPI network was generated consisting of 2973 nodes (Proteins) and 5297 edges illustrating the interaction between these genes. To focus on key regulators of this network we curated a zero-order PPI network with 49 nodes and 81 edges including only direct interaction between the seed proteins. Nodes were ranked based on the number of connections they have to other nodes (degree) and the number of shortest paths going through them (betweenness centrality) (56).

### cMAP Analysis

To find potential compounds that induce a similar or opposing gene expression pattern as our *H. pylori*-gene signature we used the Connectivity Map analysis (CMAP, https://www.broadinstitute.org/cmap/) (57, 58) as described before (59). The query yields a ranked list of various perturbagen’s signatures based on a connectivity score between − 100 to 100 where the positive scores indicate expression signature similarity between the perturbagen’s and the query signature and the negative score implies an opposing impact. The 55-gene signature (up- and down-regulated) was used to query the CMap database resulting in a connectivity score matrix of 8559 perturbations.

### Data Accessibility

All datasets used in this study are publicly available on the Gene Expression Omnibus (GEO) and ArrayExpress under the corresponding accession number. The code for this analysis is available on GitHub and can be accessed using the following link: https://github.com/Tarek-Badr/Comprehensive-Integration-of-GWAS-and-Gene-Expression-studies-in-H.pylori-induced-Gastric-Disease

## Results

### Data Acquisition

From the initial datasets acquired by searching public databases, eight matched our predetermined inclusion criteria (see *Methods*), four for human gastric biopsies – of non-cancerous origin- and four for three different cell lines. The four human gastric biopsies datasets included in the downstream analysis were used for the discovery of gene-signature and contained samples from 98 human samples, including data from 72 *H. pylori*-infected/gastritis/metaplasia patients, and 26 healthy controls. Twelve gastric cancer datasets were included for the validation process. The data summary of the included datasets is shown in **Table 1**.

**Table 1:** Summary of the datasets integrated in the meta-analysis pipeline and prediction and validation of the gene signature.

Human samples	PMID	Data set	Platform	Cell type	Controls	Cases	Refrence
1	24119614	GSE27411	GPL6255	Gastric biopsies	6	12	Nookaew et al., 2013
2	28739826	GSE60427	GPL17077	Gastric biopsies	8	24	Nagashima et al., 2015; Tanaka et al., 2017
3	28111844	GSE60662	GPL13497	Gastric biopsies	4	12	Hanada et al., 2014
4	18321301	GSE5081	GPL570	Gastric biopsies	8	24	Galamb et al., 2008
**Cell line**	**PMID**	**Data set**	**Platform**	**cell type**	**controls**	**infected**	**Refrence**
1	22889111	GSE39919	GPL6947	AGS	4	4	Kim et al., 2012
2	26802142	GSE70394	GPL6480	AGS	3	3	Costa et al., 2016
3	26690385	GSE74577	GPL17586	GES-1	3	3	Zhu et al., 2015
4	29085225	GSE74492	GPL570	HT29-MTX-E12	3	3	Cairns et al., 2017
**Tumor samples**	**PMID**	**Data set**	**Platform**	**cell type**	**controls**	**cases**	**Refrence**
1	25928635	GSE65801	GPL14550	Gastric tissue	32	32	Hao Li et al., 2015
2	24321518	E-MTAB-1440	A-MEXP-1171	Gastric tissue	20	20	Eftang et al., 2013
3	29113266	GSE79973	GPL570	Gastric tissue	10	10	Jin Y et al., 2017
4	21132402	GSE19826	GPL570	Gastric tissue	15	12	Wang, Q. et al., 2012
5	29725014	GSE13861	GPL6884	Gastric tissue	19	65	Oh SC et al., 2018
6	19081245	GSE13911	GPL570	Gastric tissue	31	38	D’Errico et al., 2009
7	24867265	GSE29272	GPL96	Gastric tissue	134	134	Li WQ et al., 2014
8	21781349	GSE29998	GPL6947	Gastric tissue	49	50	Holbrook et al., 2011
9	NA	GSE31811	GPL6480	Gastric tissue	17	21	Kitamura et al., 2011
10	22735568	GSE37023	GPL96	Gastric tissue	36	112	Wu et al., 2013
11	22735568	GSE37023	GPL97	Gastric tissue	36	29	Wu et al., 2013
12	28199974	GSE81948	GPL6244	Gastric tissue	5	15	Canu et al., 2017
**Non-gastric diseases**	**PMID**	**Data set**	**Platform**	**cell type**	**controls**	**cases**	**Refrence**
1	30653341	GSE126848	GPL18573	Liver biopsies	14	43	Suppli et al., 2019
2	NA	GSE88839	GPL570	Liver biopsies	3	35	NA
3	NA	GSE83448	GPL18134	Intestinal biopsy	14	39	NA
4	31467298	GSE130970	GPL16791	liver biopsies	6	72	Hoang et al., 2019
5	NA	GSE101685	GPL570	liver biopsies	8	24	NA
6	29782846	GSE112366	GPL13158	ileum biopsies	26	362	VanDussen et al., 2018
7	NA	GSE117999	GPL20844	cartilage tissue	12	12	NA

### Discovery and Validation of Gene Set Predictive Score Matching Cell Line Infection Models to Human Pathology

**Figure 1:**
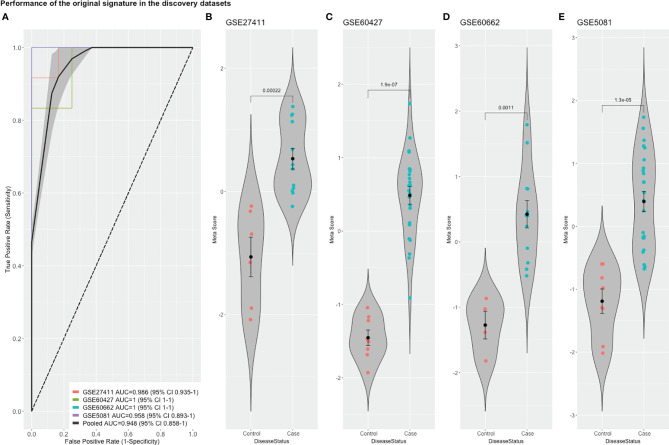
The performance of the original signature in discovery datasets. **(A)** ROC curves of the comparison between cases and controls with the pooled AUC (area under the curve) in the four training datasets. **(B–E)** violin plots of the difference in the signature meta-scores in each training dataset between cases (gastric disease) and healthy controls with each point representing a sample. Shown are p-values from Wilcoxon test.

To detect stably host-deregulated genes across various stages in *H. pylori*-induced gastritis, we compared healthy controls (controls) to samples from gastritis, atrophy, erosions, and metaplasia (cases). The initial meta-analysis resulted in the identification of 881 DEGs (294 up-regulated and 587 down-regulated genes). We refined this initial signature by using a forward search process, which resulted in the identification of 427 DEGs (225 up-regulated and 202 down-regulated genes (**Supplementary Table 1**). Our gene signature distinguished healthy controls from patients with a pooled area under the curve (AUC) = 0.948 [95% confidence interval (CI) 0.858−1] in the discovery datasets (**Figure 1A**). Violin plots of the performance of the signature in each discovery datasets shows the significant score difference between cases and controls (**Figures 1B–E**)

To match this gene signature curated from human samples to gene expression in different model cell lines, we tested the capability of the gene signature to distinguish infected from uninfected samples. The Human gene signature was able to distinguish infected samples in AGS and HT29 cell lines sufficiently with AUC = 1 in GSE39919 (AGS) and GSE74492 (E12), and AUC = 0.889 in GSE70394 (AGS) suggesting similarity of their gene signature to human gastric signature and their suitability to hypothesis testing and experimentation in comparison with human pathology (**Supplementary Figures 1A, B, D**). Interestingly our signature underperformed in distinguishing infected samples in the tested GES-1 dataset (GSE74577) (**Supplementary Figure 1C**). This was surprising: the GES-1 cells are derived from SV40 transformed human fetal gastric epithelial cells, which intuitively may be considered relatively close to primary cells (60). The gene expression analysis however suggests substantial differences to human gastric tissue. Further cell line experiments with larger sample numbers will be required to elucidate definitive similarities and differences between these *in vitro* models and the human gastric disease.

### Identification of Enriched Pathways and Upstream Signaling Activity

To understand the molecular basis and biological effect of the curated gene signature we searched for enriched pathways and gene ontologies using the KEGG and GO databases.

Unsurprisingly, immune defense related pathways and cytokine response related pathways were among the most highly enriched pathways as previously described (61). Among the most downregulated pathways were mitochondrion and mitophagy related terms, as well as various cell metabolism pathways as ATPase activator activity, mineral absorption, and folate biosynthesis.

**Figure 2:**
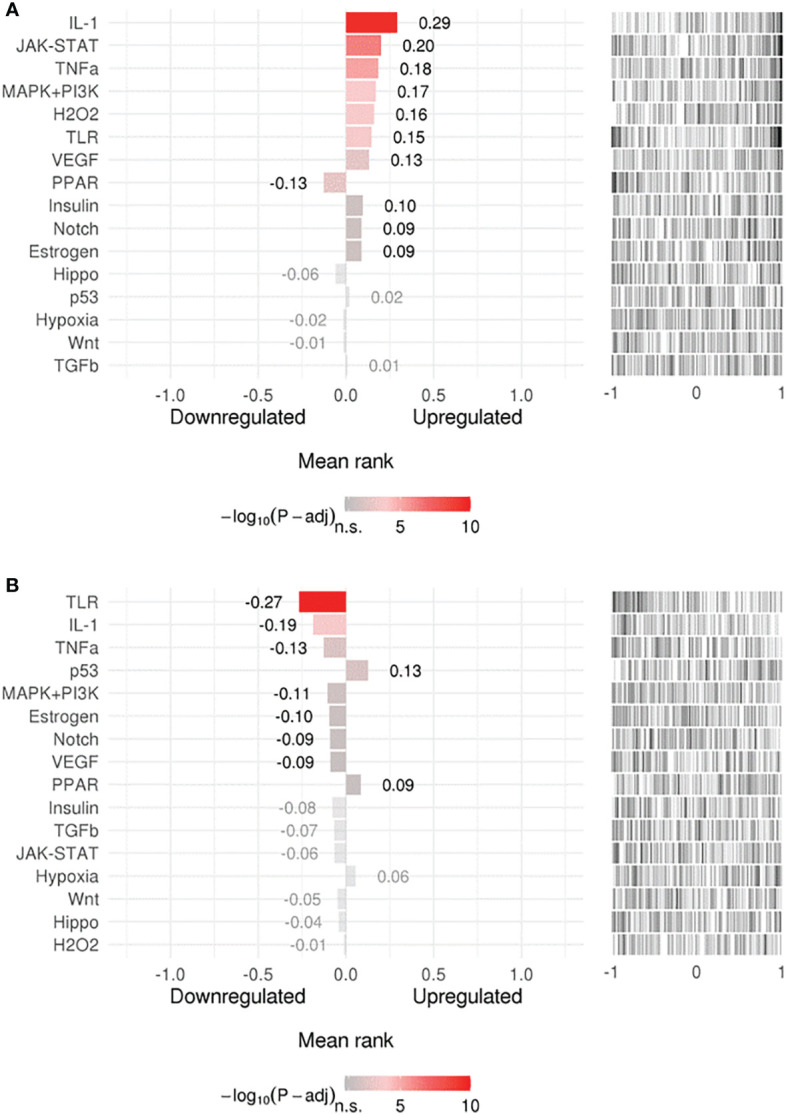
Upstream signaling pathways enrichment. Enriched upstream signals were tested in **(A)** upregulated or **(B)** downregulated genes using the (SPEED) web-tool. The x axis represents z-scores between –1 and 1 representing the rank of up- and down-regulated genes per pathway experiment. Bar graph values represent mean rank of our query gene-list for each pathway and bar colors represent adjusted p-values. Distribution of the used gene list is shown as a barcode plot on the right side of each pathway where each black bar represents a query-gene. The mean rank shift of each pathway was tested using the Bates test.

Searching for upstream signaling impact through our gene-signature through the SPEED analysis showed upregulation of IL-1, TNF, and H_2_O_2_ regulated genes (**Figure 2A**) which has been shown to induce epithelial mutagenesis (62). At the same time, this analysis permitted identification of genes known to be down-regulated by IL-1, TNF, and TLR-signals (**Figure 2B**). This is strong evidence that these pathways are up-regulated during *H. pylori*-infection. On the other hand, p53 and PPAR signaling seem to be downregulated. A list of top 10 over/underrepresented pathways in each category as well as results of SPEED analysis are shown in **Supplementary Table 2**.

### Identification of a Common Gene Expression Signature in *Helicobacter pylori-*Related Pathologies Using Random Effect Models

**Figure 3:**
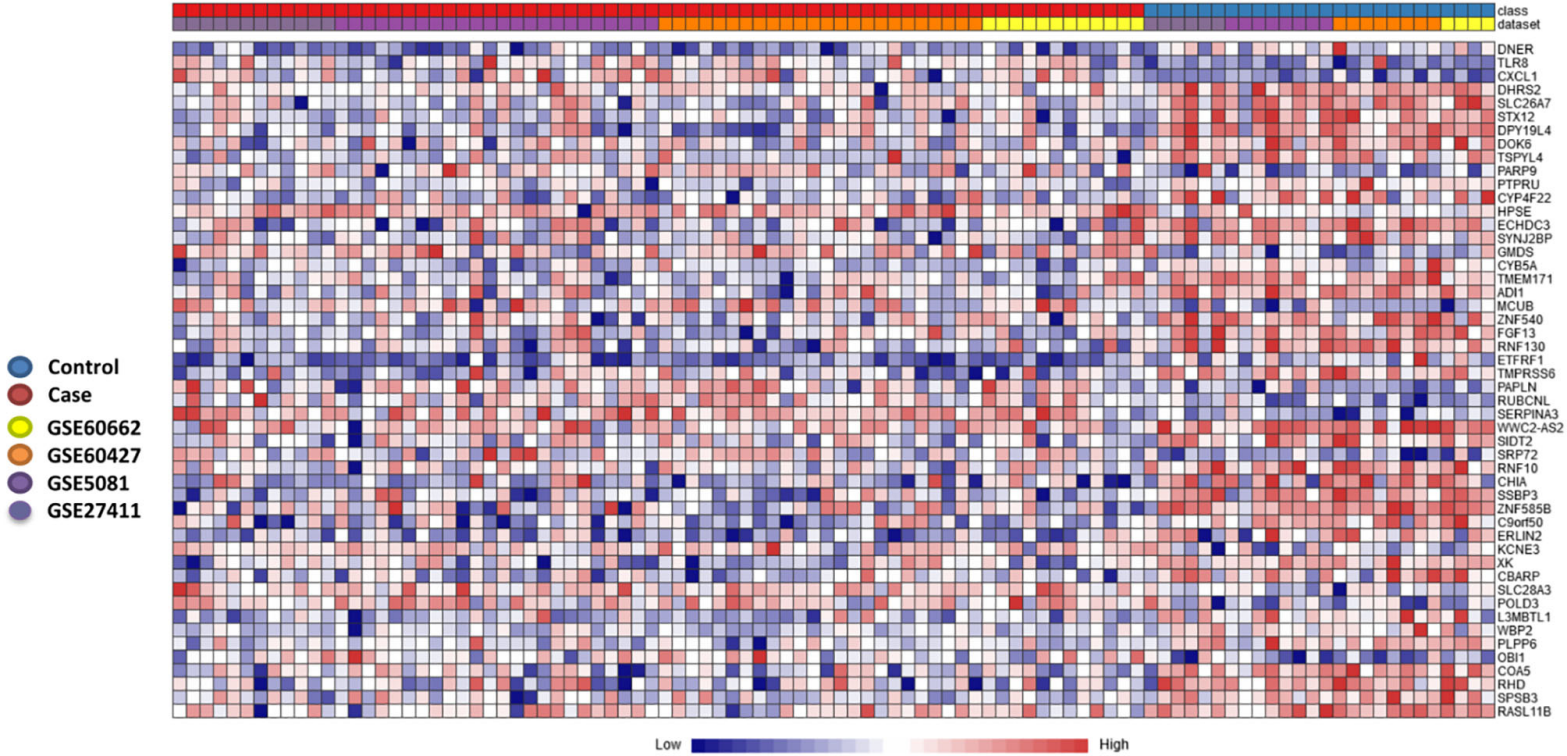
Heatmap of most significantly differentially expressed genes. Heatmap showing the relative expression of the 50 most significantly differentially expressed genes (DEGs) of the 263 significant DEGs identified through the meta-analysis, where 182 genes were co-up-regulated, and 81 genes were co-down-regulated (case versus control). The heatmap indicates the normalized expression value of each DEG in the individual samples, and genes were clustered based on their condition (cases vs controls) and their original datasets. The heatmap was created by the visualization module in NetworkAnalyst, where genes with p-value < 0.05 in the Random Effect Model analysis were considered significant.

To further stratify and refine our gene signature, we used another pipeline to determine DEGs in the discovery datasets. The individual dataset gene expression normalization was carried out using the NetworkAnalyst log2 transformation function, followed by autoscaling. The individual datasets were inspected with PCA plots before and after ComBat method normalization, and PCA plots of gene expression data of the four datasets before and after normalization and after gene expression analysis are shown in **Supplementary Figures 2** and **3** respectively. No major differences were seen that could be attributed to differences in dataset platforms or conditions and that could have introduced a bias. Based on the Cochran’s Q test analysis (**Supplementary Figure 4**) we used the REM to identify 263 genes significantly deregulated among the different human cohorts between patients and healthy controls (p<0.05 in the REM) (**Supplementary Table 3**). A heatmap of the most highly differentially regulated genes is shown in (**Figure 3**). Using this method, we see many genes identified as deregulated that were not detectable in their respective individual datasets (**Supplementary Figure 5**).

### Intersection of Gene Sets Curated Through Two Meta-Analysis Pipelines

**Figure 4:**
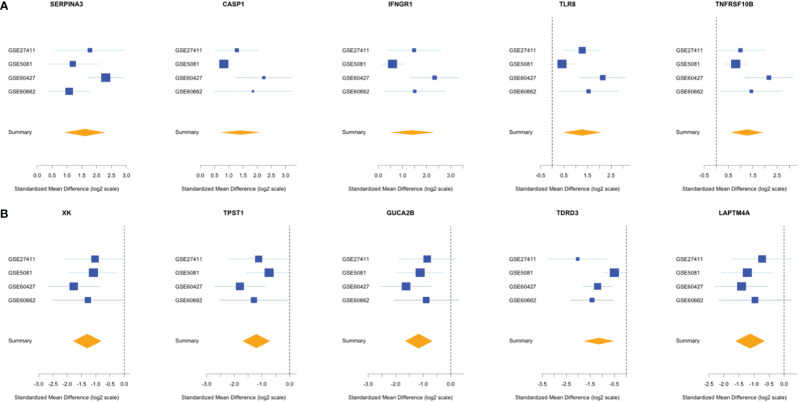
Forest plots of the 5 most up- and down-regulated genes in the intersection gene-signature. The x axis represents standardized mean difference between cases and controls for each gene. The blue rectangles’ size is oppositely proportional to the standard error of the mean in this study and their whiskers represent the 95% CI. Orange diamond represents combined mean difference of the represented gene across all studies where its width gives the 95% CI of the overall combined mean difference. **(A)** Five most upregulated genes; **(B)** five most downregulated genes.

**Figure 5:**
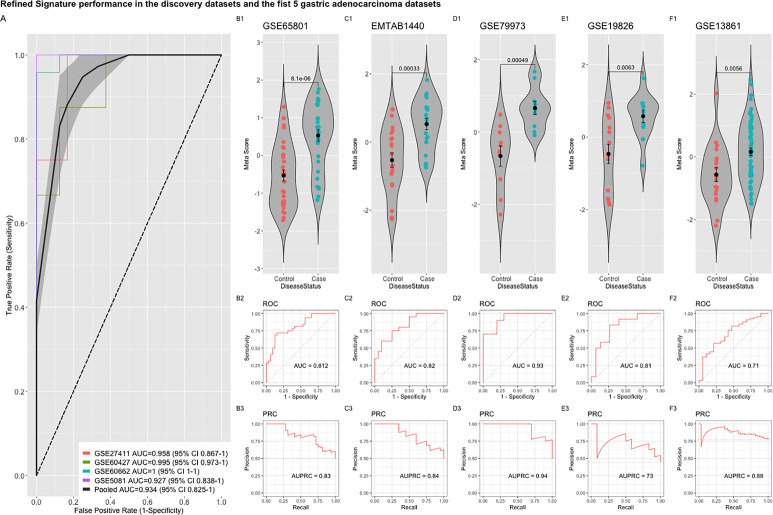
Performance of the refined 55-gene signature in the five independent gastric adenocarcinoma datasets. **(A)** The pooled AUC in the four training datasets. **(B–F)** The performance of the refined signature in the five independent gastric cancer datasets. The upper panel shows a violin plot of the difference in the refined signature meta-score between cases (gastric adenocarcinoma) and controls with each point representing a sample. The middle and lower panels show the Area under the ROC Curve (AUC) and the Area under the Precision Recall Curve (AUPRC), respectively.

Comparing this newly curated gene set with our original signature, we identified 55 genes in common between the two independent training methods; of these, 31 genes were up-regulated and 24 genes were down-regulated (**Supplementary Figure 6**). Representative forest plots of the five most up- and down-regulated genes from the 55-gene intersection signature can be seen in (**Figure 4**) and a list of the 55-gene signature can be found in **Table 2**. This refined gene signature was used for further analysis and validation. We validated this 55-gene signature in the four original discovery datasets to see if it is sufficient in distinguishing patients from healthy controls. Despite the lower gene number in comparison with the original signature and the sample heterogeneity due to dataset origin and inclusion criteria, this gene signature was able to identify patients in the four discovery datasets (Pooled AUC = 0.934 [95% CI 0.825−1]) (**Figure 5A**).

**Table 2:** Fifty-five–gene signature identified through intersection of the two meta-analysis pipelines.

Up-regulated genes	Effect Size	Down-regulated genes	Effect Size
SERPINA3	1.6036095653983	XK	-1.29819389406181
CASP1	1.40416698180637	TPST1	-1.20167450116234
IFNGR1	1.39998598913411	GUCA2B	-1.1661305569252
TLR8	1.26453608862267	TDRD3	-1.13977250937525
TNFRSF10B	1.26119688894584	LAPTM4A	-1.12730715165818
SLC28A3	1.22800817108748	DPPA5	-1.12047935329112
HPS5	1.19800916792719	SSBP3	-1.10490349676448
MLKL	1.18085464438904	CYB5A	-1.09837700706879
SNX10	1.13561882235051	UCK1	-0.985800926144472
PROS1	1.10871487093572	SS18L1	-0.96750241497866
PPA1	1.10216223136474	ADI1	-0.926114181353538
PSMB8	1.07187280747353	RAB5C	-0.84320004421934
CRELD2	1.05109963792112	RNF10	-0.842623053934248
PROK2	0.921169469208918	TSPYL1	-0.836367491510706
KCNE3	0.913103915871443	GOLPH3L	-0.824627729687288
KPNB1	0.85084526960045	CBR1	-0.771803065890106
LPIN1	0.839131410798793	LRFN3	-0.757794341085025
DGKA	0.795056386539426	NAPA	-0.740224650645346
TNIK	0.781737882009995	SLC39A1	-0.723858288394722
MCM5	0.776235448665165	PTPRU	-0.679860486124954
RCN1	0.76081302431008	KCTD1	-0.621574946697534
GMDS	0.734400543529141	NIPSNAP3B	-0.606036954338933
FCER1G	0.724174020680609	APLP2	-0.514682777690594
MICB	0.711112386548431	FBXO9	-0.383603913554028
MR1	0.659225335601429		
PARP9	0.646715982680413		
CDC42SE2	0.639975897261079		
POLD3	0.62818746016387		
HHIP	0.497135355992992		
RNGTT	0.496984121112146		
SRP72	0.443790344485934		

**Figure 6:**
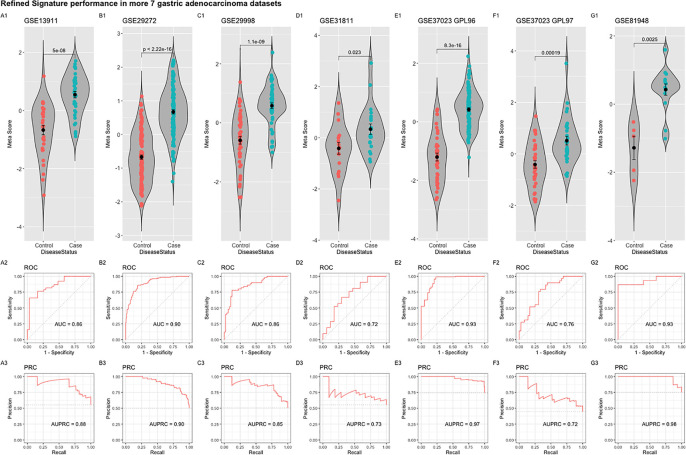
Performance of the refined 55-gene signature in the seven independent gastric adenocarcinoma datasets **(A–G)**. The upper panel shows a violin plot of the difference in the refined signature meta-score between cases (gastric adenocarcinoma) and controls with each point representing a sample. The middle and lower panels show the Area under the ROC Curve (AUC) and the Area under the Precision Recall Curve (AUPRC), respectively.

The refined 55-gene signature scored very well in distinguishing gastric cancer samples from normal tissue in all tested datasets with AUC values between (0.71–0.93). Moreover, the newly calculated meta-score of the refined signature was significantly higher in cancer samples against controls in all datasets, proving its capability in distinguishing gastric cancer tumor from controls, even when the signature comes from precancerous lesions (**Figures 5B–F**; **6A–G**). To explore the potential role of our 55-gene signature in other inflammatory diseases we tested its performance in both epithelial and non-epithelial diseases (**Supplementary Figure 7**). The gene signature underperformed in inflammatory diseases of hepatocellular origin such as fatty liver disease, liver adenoma or hepatocellular carcinoma with AUC values between 0.14 and 0.36. In other bowel inflammatory diseases such as Crohn’s disease, the signature showed a decent performance with AUC values (0.63-0.665).

### Genome-Wide Association Studies Catalog Single Nucleotide Polymorphisms Identification and Functional Annotation

Mining the NHGRI GWAS catalog database yielded 64 SNP entries associated with *Helicobacter pylori* status or gastric related pathologies. All variant related information can be found in **Supplementary Table 4**. All identified variants could be successfully annotated through the HaploReg tool resulting in 45 unique SNPs in 37 unique genes that were used for downstream analysis. From the coding variants, five were missense mutations in the genes PLCE1, CHD6, SEBOX, HABP2, and MTX1. Detailed functional analysis can be found in **Supplementary Table 5**.

### Cross-Linking Genome-Wide Association Studies With *Helicobacter pylori-*Gene Signature Through Hub Genes Network Analysis

**Figure 7:**
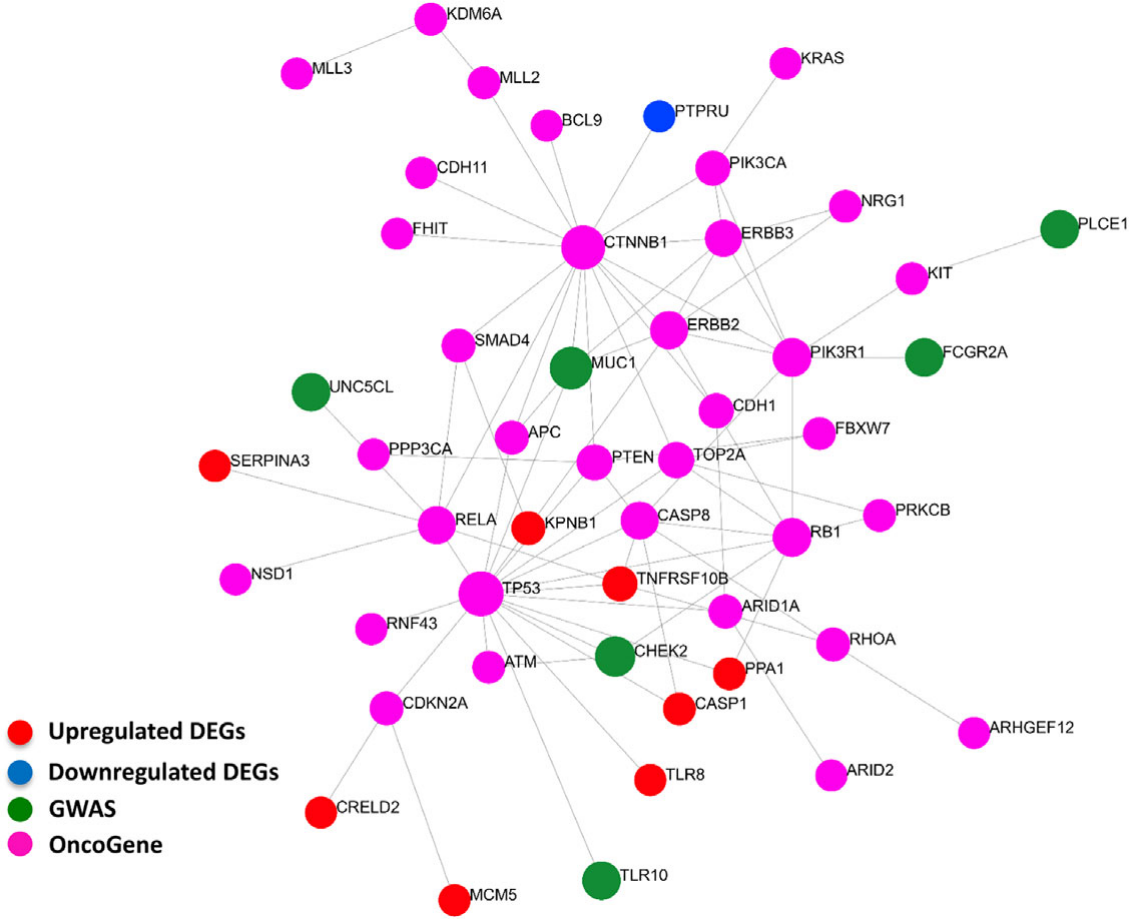
Interactions between signature derived genes and genome-wide-associated and cancer driver genes. The genes of the 55-gene signature were integrated with 37 GWAS and 61 cancer driver genes in NetworkAnalyst tools to visualize gene interactions. A “zero order” interaction network with 49 nodes and 81 edges was used. The most highly ranked nodes across the dataset based on network topology measures were TP53 (betweenness centrality = 468.99), and CTNNB1 (betweenness centrality = 450.38). TNFRSF10B (betweenness centrality = 32.53) ranked the highest among the gene signature and MUC1 (betweenness centrality = 43.66) among GWAS genes. (Red = up-regulated DEGs; Blue = down-regulated DEGs; Green = GWAS; Purple = OncoGene).

Our curated 55-gene signature represents the downstream effect of the *Helicobacter*-induced pathology. Linking it with upstream causal and cancer driver genes will be of great benefit to understand the regulation network of this signature and the interactions between its players. We performed a network-based analysis to investigate the interaction between the 55-gene signature, genes harboring polymorphisms associated with *Helicobacter* and gastric pathologies, and known gastric cancer driver genes. This analysis identified key hub genes among the most highly deregulated genes (**Figure 7**). The tumor suppressor gene tumor protein p53 (TP53) has the largest interaction with other cancer driver and downstream deregulated genes. Many of the deregulated genes seem to be directly connected to GWAS or cancer driver genes such as TLR8, CASP1, and TNFRSF10B. The data suggest that the genes that are deregulated in *H. pylori*-infection are linked to the activation of oncogenes.

### Drug Targeting of the 55-Gene Signature

As the 55-gene signature remains relevant during different stages of the disease, it was interesting to test its potentiality as a target for therapeutics development and drug repurposing. We used the signature to feed the connectivity map tool to search for compounds that are negatively correlated with this gene signature, implying their capability of inducing a reverse gene signature. Two compound classes were especially negatively enriched with scores lower than -90 hinting to their potential in opposing the *H. pylori* gene signature, which are Bromodomain Inhibitors and Leucine rich repeat kinase inhibitors. Apart from these two classes, dihomo-gamma-linolenic acid (DGLA) was one of agents inducing the highest reverse signature with a score of -92.93. Results of the highest opposing compounds and classes can be found in **Supplementary Table 6**.

## Discussion

*H. pylori* is the main cause of gastric cancer worldwide (63) and remains the only bacterium that is classified as a definite group 1 carcinogen by the World Health Organization’s (WHO) International Agency for Research on Cancer (IARC) (64). Eradication of *H. pylori* in patients and high risk carriers remains the most successful method in preventing development of gastric cancer (65). As the rates of *H. pylori* antibiotic resistance increase, the WHO has published its first ever list of antibiotic-resistant “priority pathogens”, a catalogue of 12 families of bacteria including *H. pylori* that pose the greatest threat to human health. The list was drawn up in a bid to guide and promote research and development (R&D) of new antibiotics.

An approach that has become possible through the availability of large datasets and modern computational methods is the analysis of gene regulation networks that drive disease progression and that therefore may be targets of prevention and therapy. In other infections, this approach has proved successful: using machine learning models and multi-cohort analysis it has been possible to identify global host gene expression signatures that can be used as a diagnostic framework in different diseases such as tuberculosis and Severe Dengue (66, 67).

Through our multi-cohort analysis approach, we identified a robust 55-gene signature that defines *H. pylori-*induced pathologies and that, intriguingly, remains relevant throughout disease progression to cancer. Our results show the importance of such in silico approaches to refine and polish results from heterogenic backgrounds with regard to technology and sample cohorts. Our approach could detect many genes that were underperforming in their individual datasets as TLR8. This 55-gene signature can be the basis for future pathophysiological and molecular studies for *H. pylori* induced gastritis and gastric carcinoma.

Through this signature, we were further able to compare gene expression patterns of *H. pylori* cell line infection models with the human signature. This comparison showed the expected suitability of the gastric AGS cell lines to *H. pylori* research. Gene expression profiles in the colon HT29-MTX-E12 cell line suggest that this cell is also a good model for this infection. The underperforming of the GES-1 cell line indicates the need for further validation of its response to infection and a robust control of batch effects between different labs. Such heterogeneities between different laboratories cell line batches have been recently demonstrated in the Hela cell line (68).

Detection of pathogen-associated molecular patterns’ (PAMPs) through TLRs or other receptors is a particularly important step in alerting the immune system, and this is also highlighted in our upstream-signaling SPEED analysis. *H. pylori* belongs to the Gram-negative bacteria and has LPS, which is typically recognized with TLR4, yet it manages to evade this detection through its evolved structure (69). Other TLR receptors such as TLR2 or TLR5 play a bigger role in *H. pylori* alert system (70). Our curated original and refined gene signatures emphasize a rather intriguing high upregulation of the endosomal receptor TLR8 across different samples and stages of the pathology. *H. pylori* RNA recognition and MyD88-dependent cytokine induction through TLR8 was before described (71, 72) which makes it a candidate for more extensive studies.

Caspase-1 (CASP1) can be activated through Nod-like receptors to induce cytokine production and inflammasome activation during *H. pylori* infection mainly through control of IL-1β and IL-18 levels (73). CASP1 was consistently upregulated in our two meta-analysis pipelines and appeared to influence the gene-gene interaction network through interaction with the tumor suppressor gene TP53. It has been previously shown that CASP1 transcription can be targeted through TP53 (74). This suggests an interesting basis of host background interaction with *H. pylori* pathogenesis, where TP53 modulates expression levels of CASP1, which in return can modulate *H. pylori* immune response.

The positive enrichment of cytokine-mediated signaling and response to interferon-gamma and tumor necrosis factor pathways –pathways that are commonly activated in *H. pylori* infection- in our predicted gene signature supports its biological relevance in disease pathogenesis. Interestingly, the SPEED analysis indicated an upregulation of JAK and STAT upstream signaling which was recently shown to be a coping mechanism of *H. pylori* to block IFNG signaling through reduction of cholesterol levels in infected cells to allow it to evade the host’s immune response (75). The upregulation of various genes related to C-type lectin receptor signaling pathway as FCER1G, PSMB8, and MALT1 also hints to its importance in *H. pylori* detection. The C-type lectin innate immune receptors (CLRs) can recognize various pathogen-related carbohydrate structures (76) but the spectrum of their role in *H. pylori* recognition and the extent of their involvement in gastritis development is not fully understood. CLRs have been very recently shown to be able to detect host metabolites modified by *H. pylori* and induce gastritis (77).

*H. pylori* can induce various complications but its effect on nutrition through decreasing absorption of various nutrients is especially important in children (78, 79). The downregulation of pathways related to the cellular response to zinc and copper ions as well as to mineral absorption and folate biosynthesis in our analysis paint the picture of a bacterium that can actively affect the host’s nutrition status. This validates previous recommendations of screening of *H. pylori* in diseases other than gastritis such as metabolic syndrome (80).

Dihomo-gamma-linolenic acid (DGLA) was one of agents inducing the highest reverse signatures in our connectivity map analysis. High serum values of γ-linolenic acid were associated with reduced risk for atrophic gastritis (81), and this metabolite has been considered as an anti-inflammatory and anti-proliferative agent (82). *H. pylori* converts host’s cholesterol into cholesteryl glucosides that has been recently shown to modulate host’s immunity and gastritis (77).

Some sterols such as vitamin D may be capable of competing with cholesterol to attenuate this effect (83) and fatty acids such as DGLA can be hypothesized to induce its reverse signature through a similar mechanism. The capability of DGLA to attenuate LDL uptake and to improve mitochondrial biogenesis can be hypothesized to contribute to its activity against *H. pylori-*induced gastritis as various mitochondrial viability genes vital for mitophagy regulation (e.g., TOMM7) were downregulated in our analysis (84, 85). Further validation of DGLA derivatives potential in reducing gastritis can be of great value as they can be suitable candidates for supplementary treatment in gastritis.

Various inflammatory and immune signals can be shared between tissues especially those of similar origins and in response to similar stimuli. To better understand the applicability of our 55-gene signature to other disease settings we tested its performance in other representative inflammatory diseases of epithelial origin such as Crohn’s disease, and other inflammatory diseases of non-epithelial origins such as osteoarthritis and fatty liver disease. Although the gene signature performed poorly in inflammatory and cancer diseases of the liver -which indicates major differences in the host response in these situations- it performed moderately well in other inflammatory bowel diseases indicating some shared mechanisms between the cell responses in these conditions in comparison to its response to *H. pylori* infection. Nevertheless, the cross activation of some of the components of our curated signature in other inflammatory diseases due to conserved mechanisms in epithelial cell signaling in inflammation does not undermine the relevance of the whole signature for *H. pylori-*induced pathologies, and its capability when searching for compounds inducing reverse gene signatures. In addition, the 55-gene signature remained highly sensitive in inferring disease status when tested in the context of *H. pylori* infected tissue and gastric cancer.

In this study, we identify a set of genes that remain robustly relevant for *H. pylori-*induced pathologies across different stages of the disease up to the development of gastric cancer. While only a minority of *H. pylori-*infected patients will develop cancer, the validation of our signature in cancer patients is strong support for the pathological contribution of the infection to the transformation process, while other factors such as host genetic background would complement this pathogenic effect of the disease to develop cancer. Therefore, further dissecting this signature and investigating its related pathways will illustrate the mechanisms of *H. pylori-*induced mutagenesis, and the results can be used to develop new therapeutics that counter this effect in patients with higher risk or failed eradication trials for *H. pylori*.

It is clear that this gene signature has to be further validated experimentally in larger cohorts of patients’ samples and cell lines infection models. We believe however that it can serve as a basis for further investigation of new molecular pathways and mechanisms involved in *H. pylori* pathogenesis and can help refine the results curated in them.

## Conclusion

Our study shows that the approach of a multi-cohort analysis increases sensitivity and permits the identification of candidate genes and mechanisms that may play a role in the pathogenesis of *H. pylori* associated disease including tumorigenesis. The identification of genes and pathways previously implicated by experimental studies in gastric disease in the past provides validation of the approach. Novel targets and therapeutic candidates were identified that may provide a basis for future functional and epidemiological studies. Our observations provide robust data about the underlying biology of the host response to *H. pylori* and emphasize the importance of early screening in various other diseases such as metabolic syndrome. This work could guide efforts to find new agents for prevention and therapy of gastric ulcer and cancer, especially at a time when *H. pylori* antibiotic resistance is on the rise.

## Data Availability Statement

The datasets presented in this study can be found in online repositories. The names of the repository/repositories and accession number(s) can be found in the article/**Supplementary Material**.

## Author Contributions

MTB conceived the study and the experiments. MTB and MO conducted the analyses. MTB, MO, and GH interpreted the results and wrote the paper. All authors contributed to the article and approved the submitted version.

## Funding

MTB is supported by the IMM-PACT-Program for Clinician Scientists of the Deutsche Forschungsgemeinschaft (DFG, German Research Foundation) – 413517907.

## Conflict of Interest

The authors declare that the research was conducted in the absence of any commercial or financial relationships that could be construed as a potential conflict of interest.

## Supplementary Material

Supplementary Table 1 Summary of 427 DEGs identified by MetaIntegrator pipeline analysis.

Supplementary Table 2 List of top 10 over/underrepresented pathways and gene ontologies as well as SPEED analysis.

Supplementary Table 3 Summary of DEGs identified in individual datasets through limma and combined meta-analysis through NetworkAnalyst pipeline.

Supplementary Table 4 Helicobacter-associated pathologies risk variants identified by GWAS analysis.

Supplementary Table 5 Function analysis of identified SNPs.

Supplementary Table 6 List of top 50 compounds and compound classes inducing reverse signature to the *H. pylori* 55-gene signature in CMAP analysis.
