# Supplementary material for: Comprehensive Integration of Genome-Wide Association and Gene Expression Studies Reveals Novel Gene Signatures and Potential Therapeutic Targets for Helicobacter pylori-Induced Gastric Disease
Source: Front Immunol. 2021 Feb 24;12:624117. doi: 10.3389/fimmu.2021.624117 (PMC7945594; doi:10.3389/fimmu.2021.624117)
Supplement: Supplementary file 7 [file DataSheet_1.pdf]

PRISMA 2009 Flow Diagram

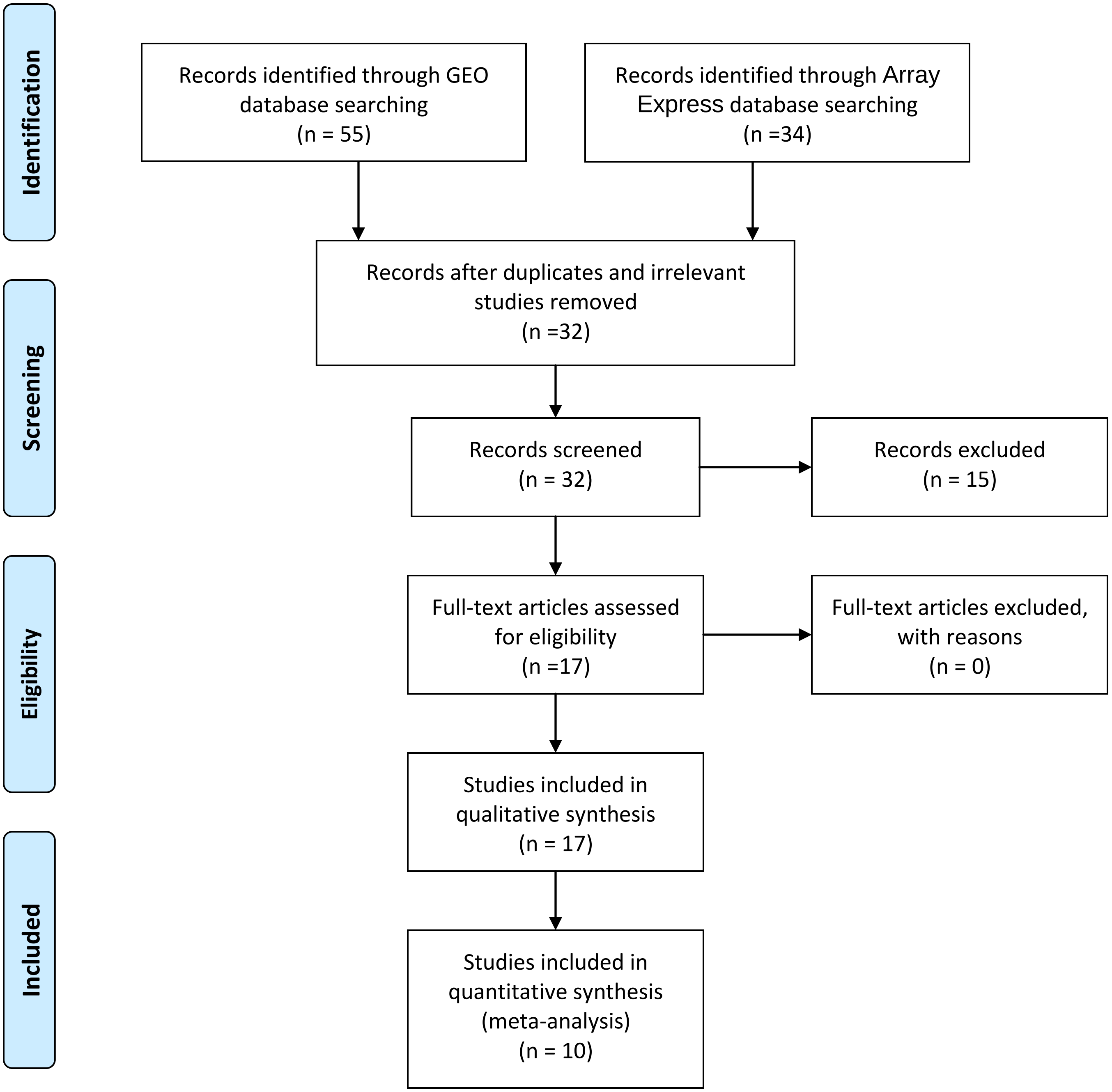

From: Moher D, Liberati A, Tetzlaff J, Altman DG, The PRISMA Group (2009). *P*referred *R*eporting *I*tems for *S*ystematic Reviews and *M*eta-*A*nalyses: The PRISMA Statement. PLoS Med 6(7): e1000097. doi:10.1371/journal.pmed1000097

For more information, visit www.prisma-statement.org.
