# Supplementary material for: Comprehensive Integration of Genome-Wide Association and Gene Expression Studies Reveals Novel Gene Signatures and Potential Therapeutic Targets for Helicobacter pylori-Induced Gastric Disease
Source: Front Immunol. 2021 Feb 24;12:624117. doi: 10.3389/fimmu.2021.624117 (PMC7945594; doi:10.3389/fimmu.2021.624117)
Supplement: Supplementary file 8 [file DataSheet_2.pdf]

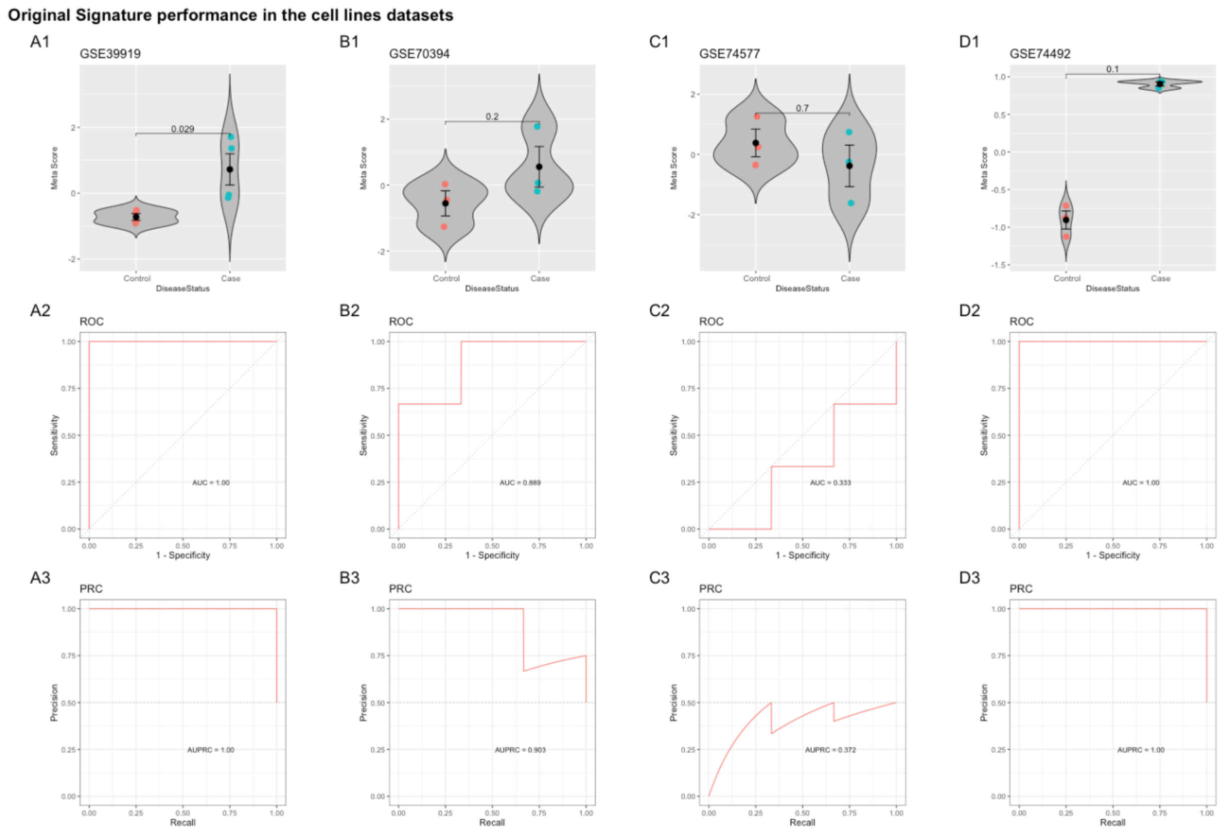

Supplementary Figure 1. The performance of the original signature in the four cell lines datasets

A) The performance of the original signature in the gastric cell line AGS dataset (GSE39919). B) The performance of the original signature in the AGS cell line dataset (GSE70394). c) The performance of the original signature in the GES-1 cell line dataset (GSE74577). d) The performance of the original signature in the E12 cell line dataset (GSE74492). In each, the upper panel shows a violin plot of the difference in the signature meta-score between cases (gastric adenocarcinoma) and controls with each point representing a sample. The middle and lower panels show the Area under the ROC Curve (AUC) and the Area under the Precision Recall Curve (AUPRC), respectively.

A. B.

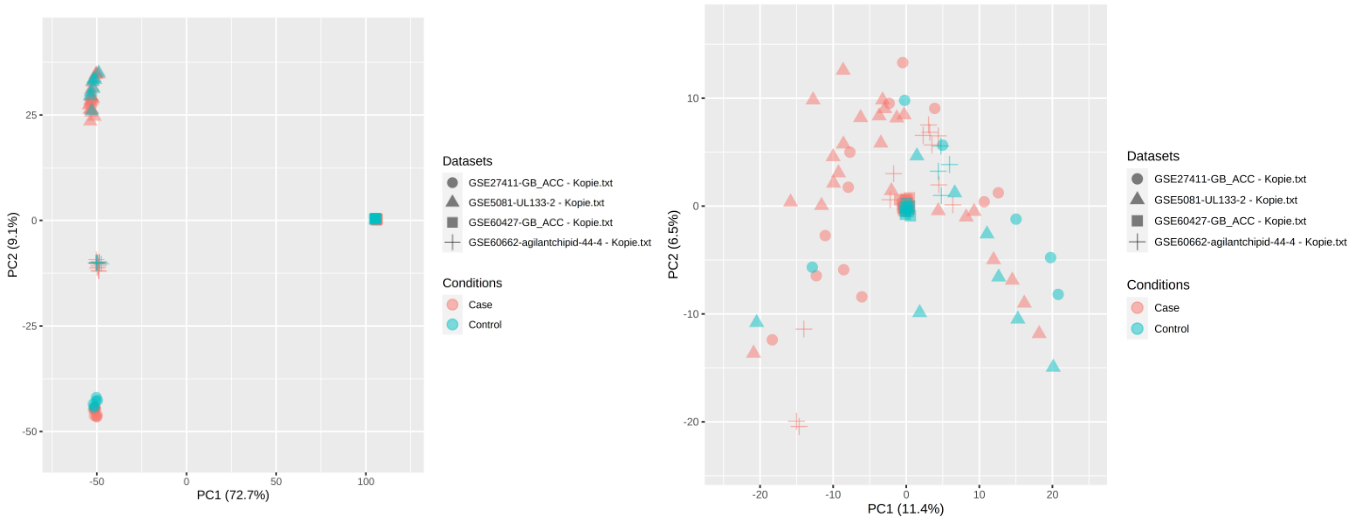

Supplementary Figure 2. PCA plots of included datasets gene expression in NetworkAnalyst before and after normalization.

The PCA plots of the four datasets included in the meta-analysis before (a) and after batch effect adjustment (b) using the ComBat batch effect method. The PCA plots are shown based on spanning of their first two principal components.

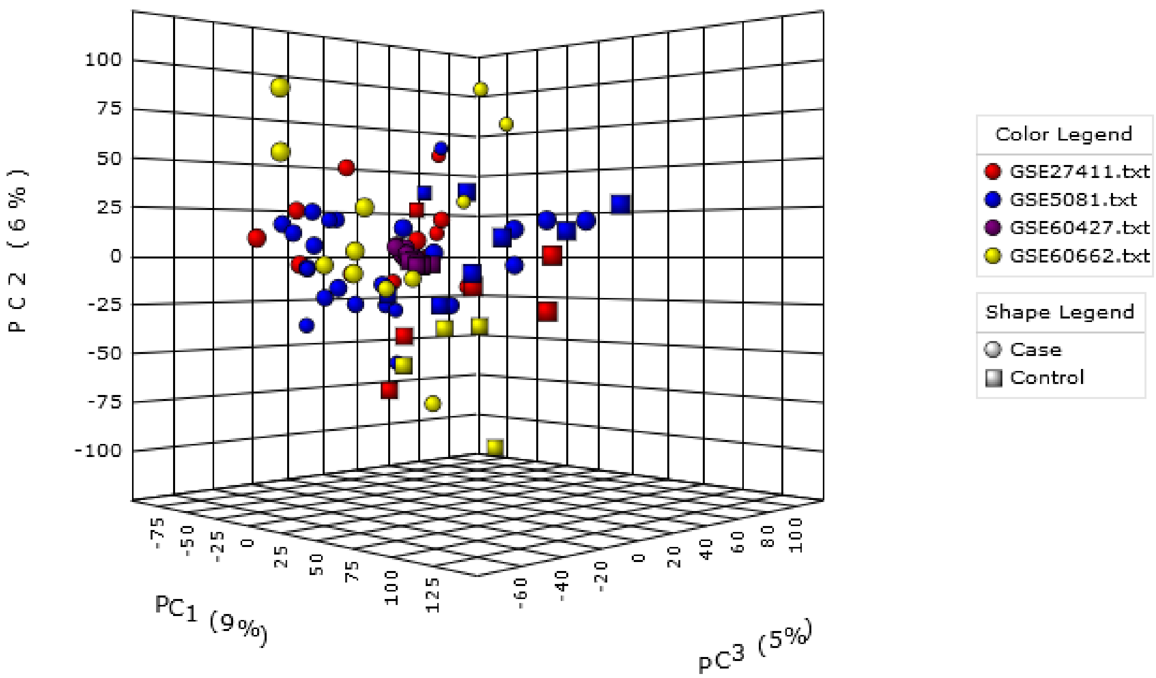

Supplementary Figure 3. PCA plot of training datasets based on their gene expression.

The PCA plot of the 4 datasets in the 3D plane is shown based on spanning of their first three principal components. No major differences that can be attributed to dataset specific platform or variable biases were identified.

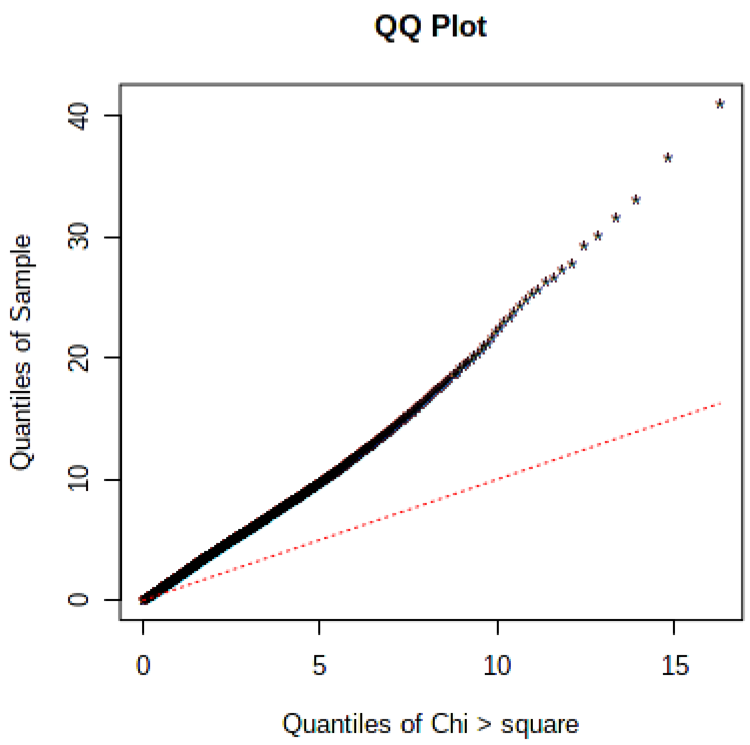

Supplementary Figure 4. Quantile-Quantile plot of the Cochran's Q test.

The Quantile-Quantile plot of the Cochran's Q test shows deviation of the Q values from the chi-squared distribution. Based on the Cochran's Q test graphs implementation of the Random Effect model was the appropriate model to analyze the included datasets in the meta-analysis.

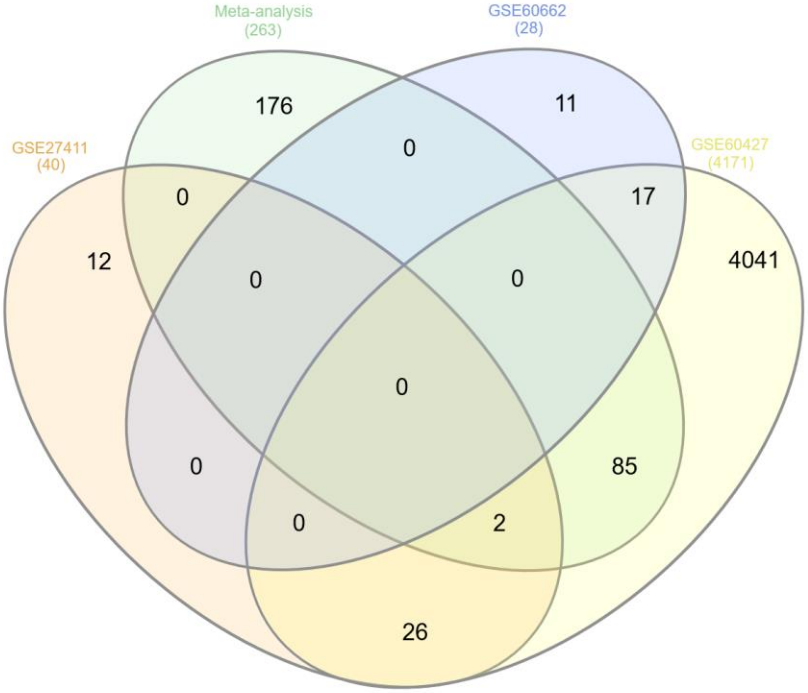

Supplementary Figure 5. Venn diagram of DEGs from limma individual datasets analysis and NetworkAnalyst meta-analysis.

In comparison with the analysis of individual datasets the combined meta-analysis shows many DEGs (176) that only were significantly different following this approach. Loss of genes that were only significant in their respective disease datasets (genes that play no role common to both conditions) is also expected. Data sets were analyzed with the same parameters in NetworkAnalyst, and genes with a p-value < 0.05 were considered significant.

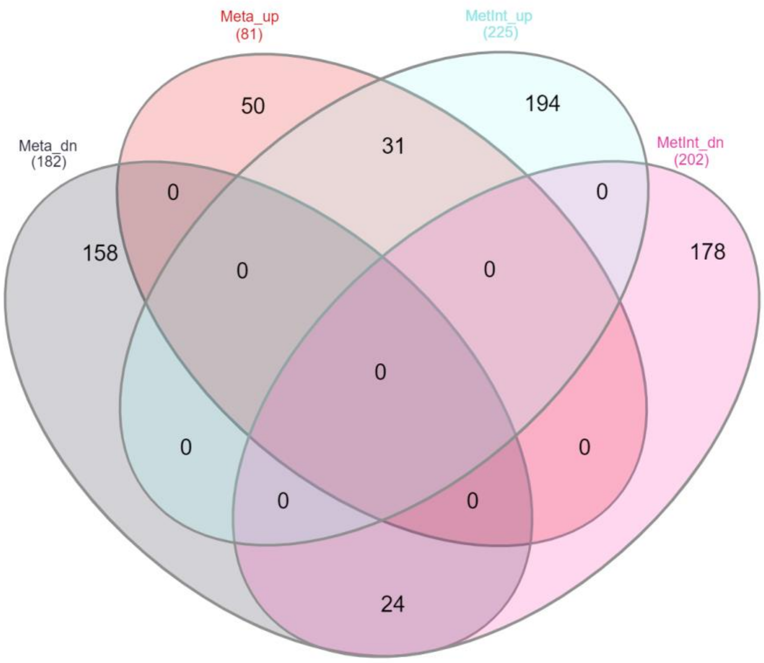

Supplementary Figure 6. Intersection of the two gene signatures from MetaIntegrator and NetworkAnalyst. Using two different meta-analysis methods we identified 55 genes in common between the two gene sets; of which 31 genes were up-regulated and 24 genes were down-regulated. Meta = signature identified by NetworkAnalyst; Metint = Original signature identified by MetaIntegrator.

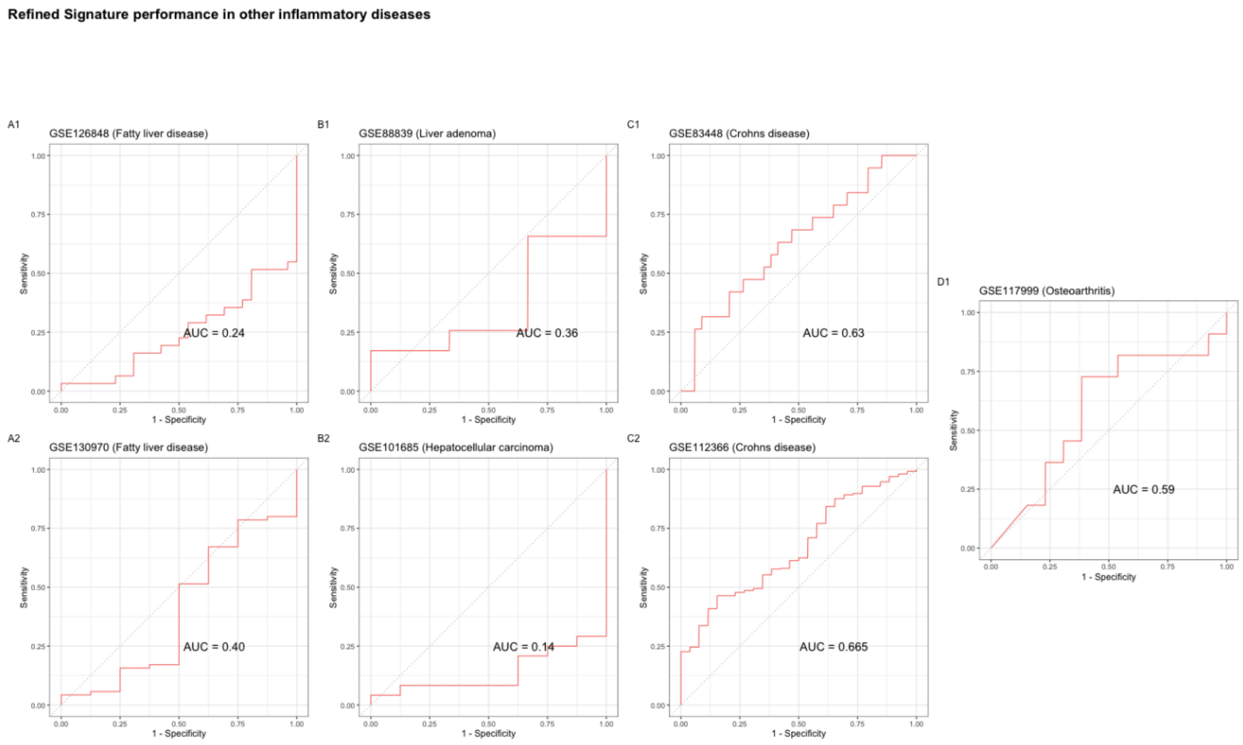

Supplementary Figure 7. The performance of the 55-gene signature in the other inflammatory diseases datasets

The performance of the 55-gene signature in other non-gastric inflammatory and cancer diseases. The plots show the Area under the ROC Curve (AUC).
